# Pulmonary Tumor Thrombotic Microangiopathy With Versican Expression in a Patient With Advanced Gastric Cancer: A Case Report of a Rare Autopsy

**DOI:** 10.7759/cureus.65690

**Published:** 2024-07-29

**Authors:** Satoru Muramatsu, Masaya Fujiwara, Kurata Kazunari, Masatoshi Watanabe, Noriko Watanabe

**Affiliations:** 1 Department of Pathology, Mie University Hospital, Tsu, JPN; 2 Department of Pathology, Mie Chuo Medical Center, Tsu, JPN; 3 Department of Gastroenterology, Mie Chuo Medical Center, Tsu, JPN; 4 Department of Pathology, Mie University Graduate School of Medicine, Tsu, JPN

**Keywords:** versican, autopsy, respiratory failure, gastric cancer, pulmonary tumor thrombotic microangiopathy

## Abstract

Pulmonary tumor thrombotic microangiopathy (PTTM) is a rare but fatal complication of a malignant tumor that causes rapidly progressive pulmonary hypertension (PH). We report the case of a 70-year-old Japanese man who died of respiratory failure during chemotherapy for gastric cancer and was diagnosed with PTTM at autopsy. The autopsy revealed PTTM-specific histological findings, such as tumor emboli with fibrin-rich clots and fibrocellular intimal proliferation in the vessels. The cancer cells were immunohistochemically positive for vascular endothelial growth factor and platelet-derived growth factor, whereas the thickened intima of the pulmonary arteries was positive for versican (VCAN). As VCAN is an extracellular matrix proteoglycan that is dramatically increased in vascular lesions of pulmonary arterial hypertension, this case demonstrates that VCAN is also involved in the pathophysiology of PTTM.

## Introduction

Pulmonary tumor thrombotic microangiopathy (PTTM) is a rare condition associated with neoplastic disorders that leads to pulmonary hypertension (PH), right-sided heart failure, and a poor prognosis [1]. PTTM was first reported in 1990 by von Herbay et al. [2]. Most cases of PTTM originate from adenocarcinoma, predominantly gastric cancer, and are diagnosed at autopsy. PTTM is found in 3.3% of autopsy cases in patients with malignancy, but as high as 16% in patients with gastric cancer. Ante-mortem diagnosis is rare and difficult because of the rapid progression of PH, right-sided heart failure, and death [1].

Platelet-derived growth factor (PDGF) and vascular endothelial growth factor (VEGF) produced by tumor cells are considered to be important factors involved in fibrocytic proliferation of the vascular intima [3]. Versican (VCAN) is an extracellular matrix proteoglycan that is present in small amounts in normal blood vessels, but it is known to increase dramatically in vascular lesions, suggesting the involvement of VCAN in pulmonary vascular remodeling [4].

Herein, we report the case of a 70-year-old Japanese man who died of respiratory failure during chemotherapy for gastric cancer and was diagnosed with PTTM at autopsy. We also showed VCAN expression in the matrix of tumor emboli and in the intima and media of small pulmonary arteries with tumor emboli, as well as VEGF and PDGF expression in tumor cells.

## Case presentation

**Figure 1:**
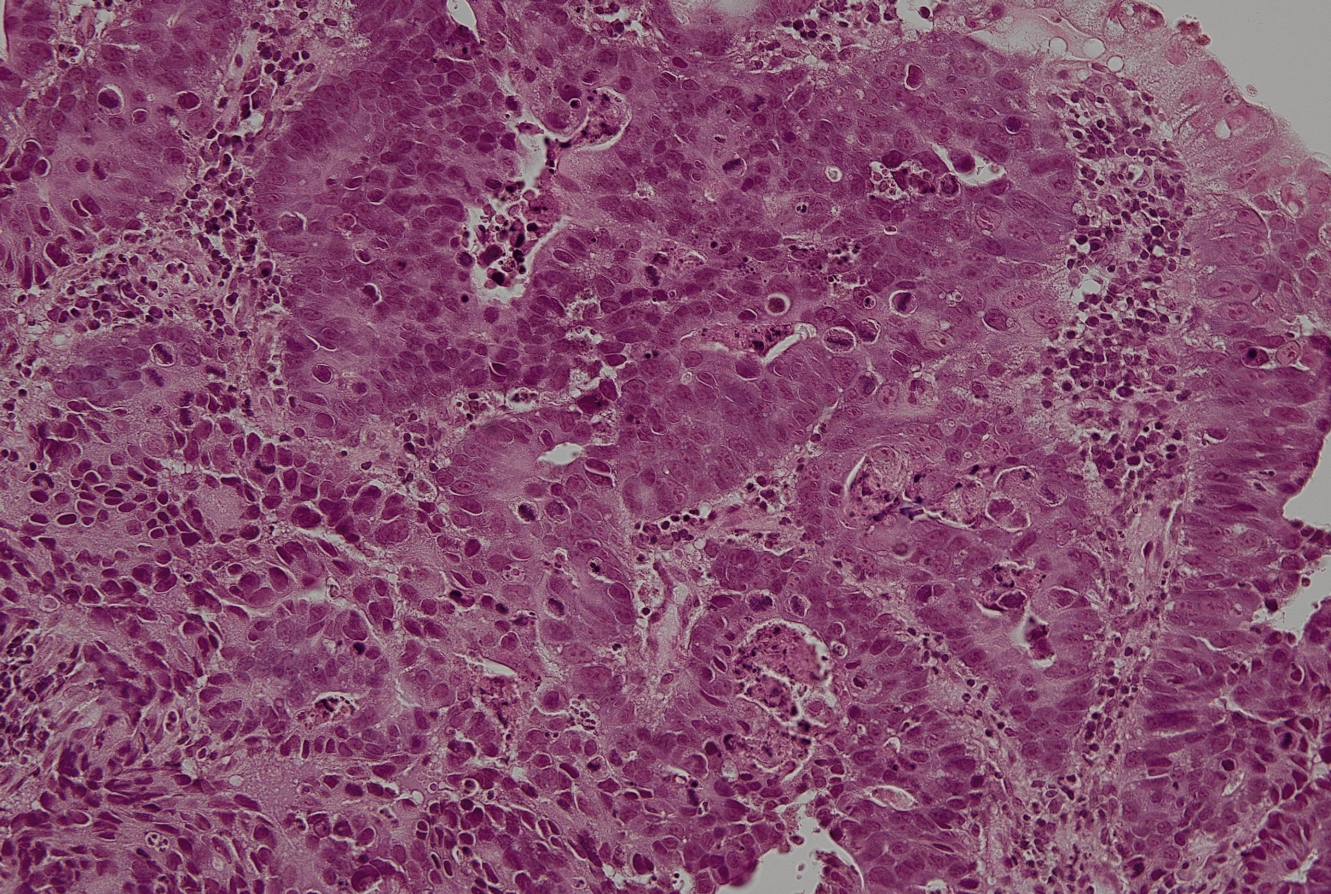
Histopathological findings of the gastric lesion on biopsy (hematoxylin & eosin staining, original magnification 20×). Moderately differentiated tubular adenocarcinoma was demonstrated.

A 70-year-old Japanese man was diagnosed with advanced gastric cancer (moderately differentiated tubular adenocarcinoma, cT3N3M1) one year ago (Figure 1). After 11 cycles of chemotherapy with tegafur-gimeracil-oteracil potassium and cisplatin (CDDP), the patient was started on outpatient chemotherapy with ramucirumab and paclitaxel three weeks before admission due to further progression of gastric cancer. The patient was admitted to our hospital with bilateral lower-extremity edema and loss of appetite. Ramucirumab and paclitaxel chemotherapy was discontinued.

**Figure 2:**
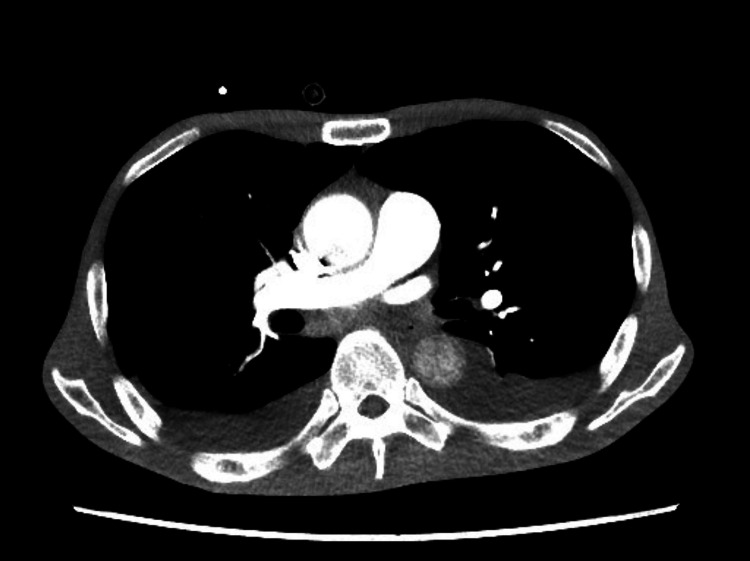
Contrast-enhanced computed tomography of the chest demonstrates bilateral pleural effusion but no pulmonary emboli.

Abdominal contrast-enhanced computed tomography (CECT) revealed gastric wall thickening, multiple swollen lymph nodes, liver and bone metastases, and thrombosis of the inferior vena cava. Chest CECT revealed bilateral pleural effusion but no pulmonary emboli (Figure 2). Anticoagulation with oral rivaroxaban for inferior vena cava thrombosis was initiated on the first day, and fever was observed from the sixth day. On the eighth day, the fever resolved; however, hematemesis and progressive anemia were observed, and anticoagulation therapy was discontinued. On the same day, oxygen administration was started owing to decreased oxygen saturation, but there was no improvement; his respiratory condition worsened, and he died on the 18th day.

**Figure 3:**
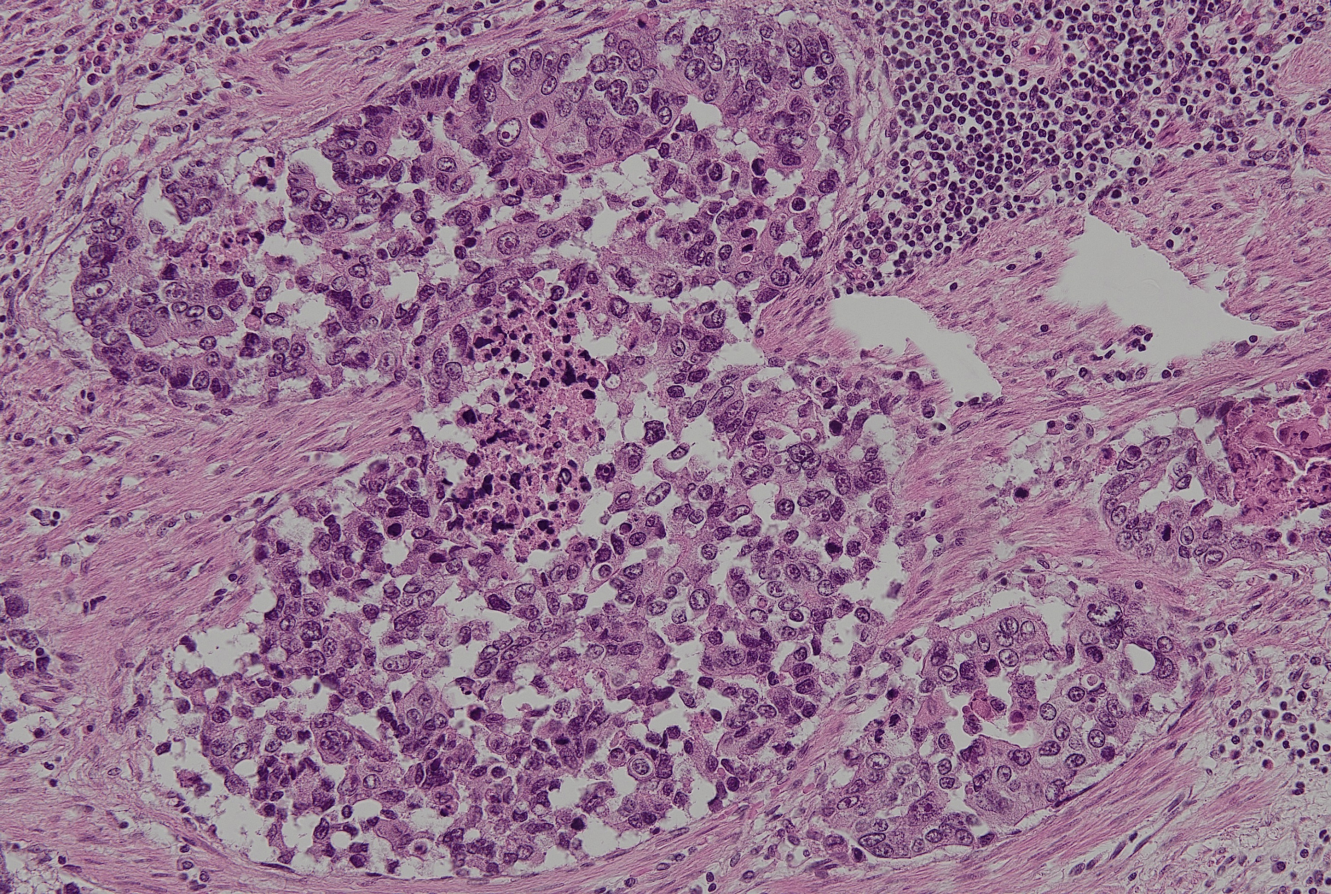
Histopathological findings of the gastric lesion on autopsy (hematoxylin & eosin staining, original magnification 20×). Poorly differentiated adenocarcinoma was demonstrated.

An autopsy was performed with the approval of the patient’s family. Macroscopically, a Borrmann type 3 lesion (85×35 mm), which formed an ulcer and had an indistinct border with the surrounding area, was observed at the greater curvature of the middle of the stomach corpus, with erosion and ulcerative and infiltrative lesions in the upper body of the stomach. Microscopically, a poorly differentiated adenocarcinoma was detected (Figure 3) that had invaded the muscularis propria and showed severe vascular invasion in the muscularis propria and subserosa. Peripancreatic, para-aortic, and perigastric lymph node metastases were detected, as well as metastases in the liver, adrenal gland, and bone marrow.

**Figure 4:**
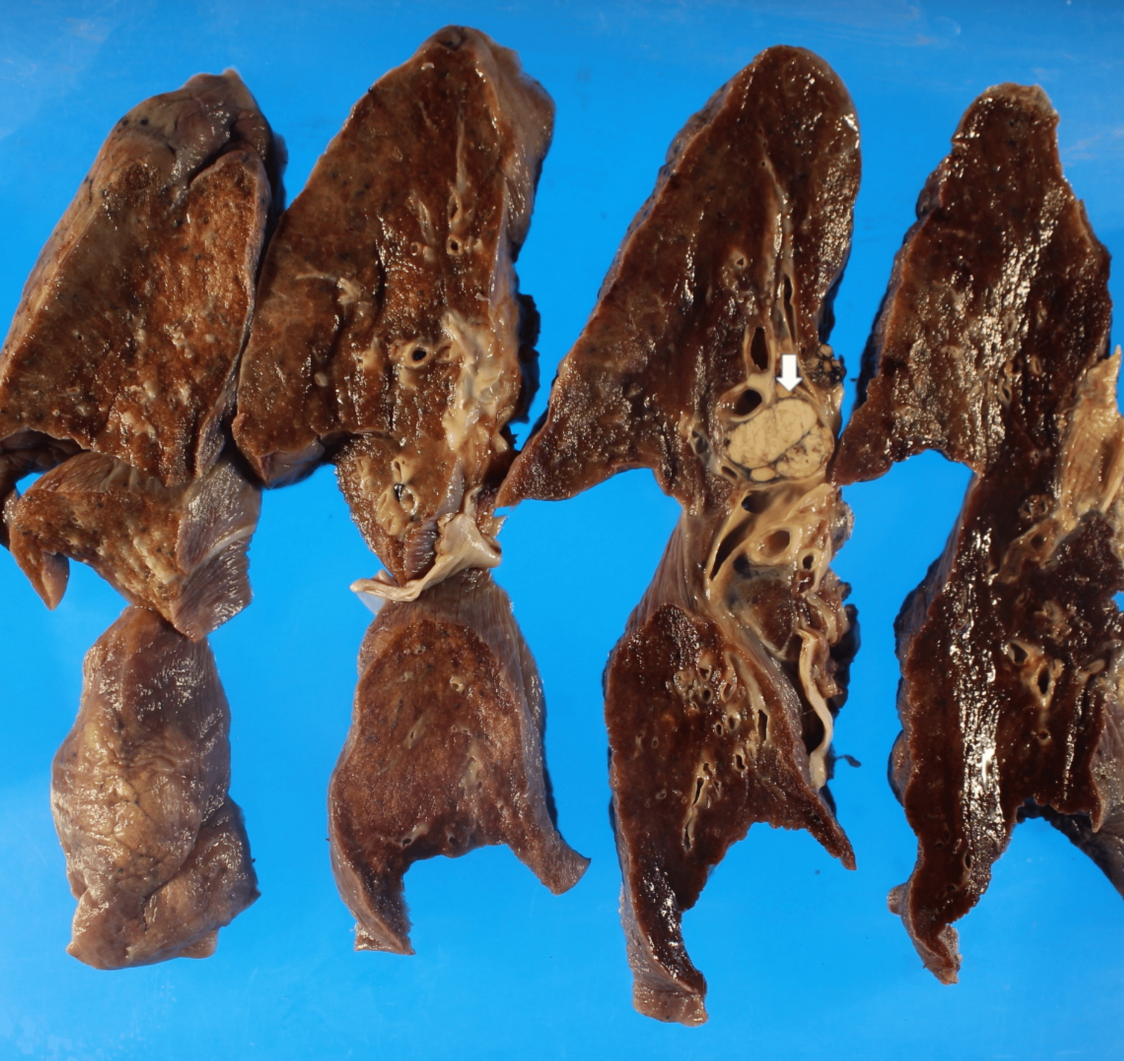
Grossly, the right lung demonstrated edematous tissue and congestion, along with metastasis in the lymph node (indicated by the white arrow).

**Figure 5:**
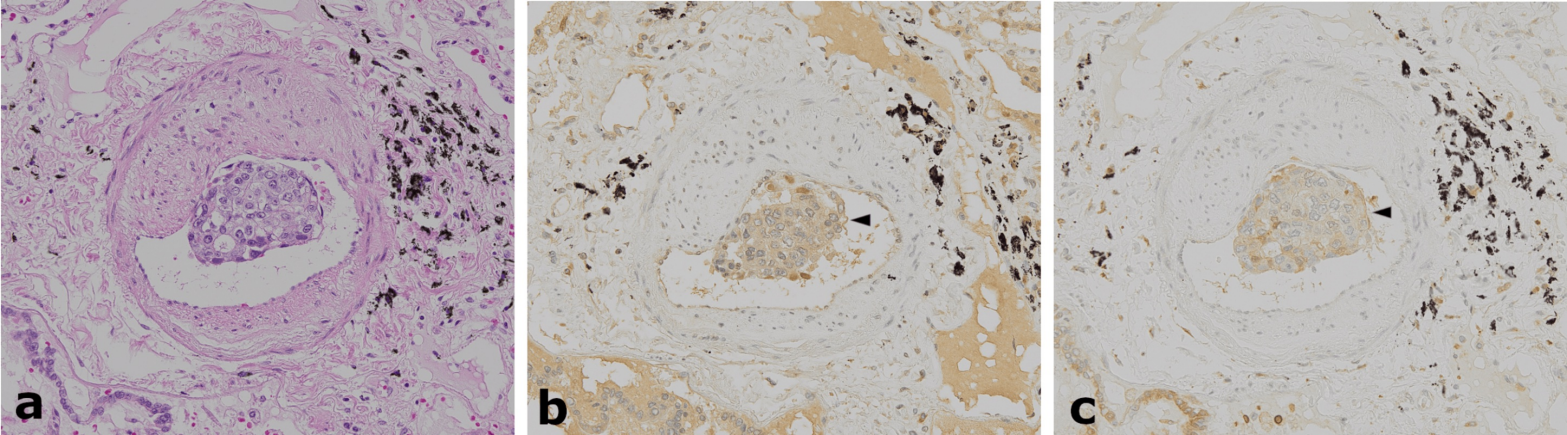
Immunohistochemical findings of the metastatic cells in the small pulmonary arteries. a) Hematoxylin and eosin staining (original magnification 20×). b) Staining for vascular endothelial growth factor (VEGF) (original magnification 20×); positive in the cytoplasm of tumor cells (indicated by black arrowhead). c) Staining for platelet-derived growth factor (PDGF) (original magnification 20×); positive in the cytoplasm of tumor cells (indicated by black arrowhead).

In the respiratory system, the weight of both lungs increased (left: 420 g, right: 515 g), with congestion and edema, and metastasis was observed in the hilar lymph nodes, but not in the lung parenchyma. No obvious thrombi were detected in the pulmonary artery or its main branches (Figure 4). Microscopically, diffuse multiple tumor emboli and clot formation were observed in the small pulmonary arteries and arterioles throughout both lungs, and fibro-cellular intimal proliferation was observed in the intima of these vessels (Figure 5). Organized thrombi and vascular recanalization were also observed. All these findings are characteristic of PTTM. No evidence of carcinomatous lymphangiosis was observed.

**Figure 6:**
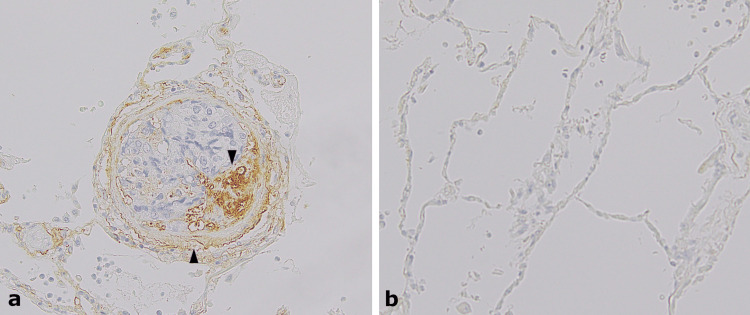
Immunohistochemical findings of versican (VCAN) (original magnification 20×). a) Versican is expressed in the cytoplasm of proliferating intimal cells and vascular smooth muscle cells (indicated by black arrowhead) but was not detected in tumor cells. b) Very weak or no expression in the alveolar wall.

The final diagnosis was PTTM owing to a poorly differentiated adenocarcinoma of the stomach. Immunohistochemical staining of the PTTM lesion was performed for VEGF using an anti-VEGFA antibody (mouse monoclonal, ab1316), PDGF using an anti-PDGF AA antibody (rabbit polyclonal, ab216619), and VCAN using an anti-VCAN antibody (rabbit monoclonal, ab270445). The expressions of VEGF and PDGF were detected in tumor cells that adhered to the vascular intima (Figure 5). VCAN was detected in the matrix of tumor emboli and within the intima and media of the small pulmonary arteries and small arterioles with tumor emboli but was only weakly expressed in the surrounding alveolar wall and not in tumor cells (Figure 6).

## Discussion

PTTM is a rare but fatal disease characterized by rapidly progressive hypoxia and PH. Ante-mortem diagnosis of PTTM is currently challenging, apart from only a few cases, including pulmonary aspiration cytopathology [5,6]. Although chemotherapy, anticoagulation, steroids, oxygen therapy, and medications used to treat pulmonary arterial hypertension (PAH) are applied, the optimal management of PTTM has not been determined. PTTM is pathologically considered the result of tumor emboli-induced coagulation cascade activation, fibrin clot formation, and fibrocellular intimal proliferation, leading to vascular luminal stenosis, mainly in the pulmonary arterioles and small arteries [1-6]. Although the mechanism is not fully understood, complex interactions between tumor cells, endothelial cells, smooth muscle cells, and inflammatory cells via cytokines and growth factors, such as VEGF and PDGF, are considered to play crucial roles in PTTM [1,7,8]. Therefore, it is important to inhibit this intimal proliferation to improve PH. Moreover, PH can be partially or completely controlled by imatinib (PDGF receptor antagonist) and bevacizumab (VEGF receptor inhibitor) [7,8]. In this case, the expression of PDGF and VEGF in tumor cells was also confirmed, and these molecular-targeted agents may have been useful. However, in PTTM with progressive dyspnea, the effects of molecular-targeted agents are limited or ineffective.

In addition, we observed VCAN expression in vascular lesions. VCAN is a large chondroitin sulfate-containing proteoglycan that interacts with hyaluronan through specific domains in its core protein and is mainly derived from smooth muscle cells and adventitial fibroblasts [4]. VCAN is expressed in many tissues during embryogenesis and at low levels in healthy adult tissues. However, VCAN is rehashed and accumulates in inflammation and mechanical stress. VCAN plays a role in wound healing and tissue remodeling [9]. For example, VCAN accumulation occurs in various human lung diseases, especially in vascular lesions in PAH [4]. PAH is characterized by vasoconstriction, wall thickening of the pulmonary arteries, and increased vascular resistance. Increased VCAN expression has also been observed in malignant tumors [9]. PAH, which is caused by PTTM, is characterized by the remodeling of the pulmonary vasculature rather than mechanical obstruction by tumor cells. However, an interesting point in our patient is that immunohistochemical staining shows no expression of VCAN in tumor cells. The signals from PDGF and VEGF stimulate the formation of VCAN-hyaluronan aggregates in the extracellular matrix and the expansion of the extracellular matrix [10]. This may result in the increased synthesis of VCAN. VCAN-hyaluronan aggregates also provide a permissive environment for arterial smooth muscle growth [4]. In addition, inhibition of VCAN blocks the proliferation of smooth muscle cells, suggesting a key role in arterial remodeling in vascular disease [11]. Thus, we also speculate that VCAN is involved in the pathogenesis of PTTM, leading to the discovery of specific antiproliferative agents.

## Conclusions

In PTTM, PH is caused by the intimal fibrocellular proliferation of the pulmonary arteries or arterioles. In this case, we confirmed the expression of VEGF and PDGF in tumor cells, and VCAN in the vascular lesions, using immunohistochemical staining. It is possible that VCAN is induced by these growth factors and contributes to the intimal thickening.
